# Kampala Commitment 2019

**DOI:** 10.4102/phcfm.v11i1.2176

**Published:** 2019-09-18

**Authors:** Shabir Moosa

**Affiliations:** 1 Department of Family Medicine and Primary Care, Faculty of Health Sciences, University of the Witwatersrand, Johannesburg, South Africa; 2 WONCA Africa, Johannesburg, South Africa

Kampala Commitment 2019: The commitment by family doctors to universal health coverage, primary health care and building the capacity of primary health care teams to scale in Africa.

We, the 181 delegates from 32 countries gathered in Kampala (04–08 June 2019) at the WONCA Africa Regional Conference on Family Medicine (including the Primafamed Pre-Conference Meeting) state the following:

- We confirm our support for the ‘Declaration of Astana’, adopted at the Global Conference on Primary Health Care in Astana (25–26 October 2018), stating that ‘… strengthening primary health care (PHC) is the most inclusive, effective and efficient approach to enhance people’s physical and mental health, as well as social well-being, and that PHC is a cornerstone of a sustainable health system for universal health coverage (UHC) and health-related Sustainable Development Goals’. We will actively support it in Africa.
- We remind the international community of the potentially explosive growth in the African population and massive urbanisation of Africa by the turn of this century based on UN Population Prospects 2017 and the need to address PHC in Africa with great urgency. Universal Health Coverage should go beyond financing to include service delivery reforms that create relevant, accessible, responsive, person-centred, quality, cost-effective and sustainable first contact care. UHC (with PHC as a priority) should incorporate a whole system approach (inclusive of non-state actors and human resources from private and non-government sectors) and payment reform with strategic purchasing.
- We believe that overly-simplistic solutions for PHC through vertical disease-based and risk-oriented approaches are dangerous, and we welcome the move towards meeting health needs across the life course of individuals with services that are integrated, continuous and people-centred.
- We remind the international community of its commitment ‘to train and retain adequate numbers of health workers, with an appropriate skill mix, including primary health care nurses, midwives, allied health professionals and family physicians, who are able to work in a multidisciplinary context, in cooperation with non-professional community health workers in order to respond effectively to people’s health needs in the World Health Assembly (WHA) resolution 62.12 (Primary Health Care, including health system strengthening, 22 May 2009).
- Whilst we are acutely aware of human and other resource challenges for PHC in Africa, we are convinced that family doctors should be located within community-based inter-professional PHC teams that may vary in the team composition and size of population enrolled by the countries. Whilst we are usually placed with other healthcare professionals in the primary care setting, family doctors actively promote community-oriented primary care (COPC), as a strong combination of personal care and public health, so that we enable the PHC team to also address social determinants of health at a population level. We commit to integrate working life in the delivery of people-centred care in line with the WONCA/International Commission on Occupational Health statement on workers’ health and their families.
- We, as family doctors, are actively developing the discipline of family medicine (the medical ‘specialty’ of primary care) as appropriate to Africa. We are actively exploring how we can contribute to strong PHC teams through expanding family medicine training for doctors and building the capability of all PHC team members. We are preparing to scale up quickly to match the needs and challenges within Africa, including ensuring that such plans are rural-proofed. The Sudan (through their distance-based masters programme), Europe (through the Expert Panel on Effective Ways of Investing in Health, advising the European Commission) and China (through the GP system) offered some very useful insights into appropriately building family doctor-led teams to scale. We urge governments to take measures to provide posts, decent working conditions and occupational services for all PHC providers.
- We are convinced that scaling up of PHC teams in Africa can only happen in a sustainable way through a concerted and integrated effort addressing PHC capacity building and migration. The consequences for the rest of the world will be grave if access and quality of primary healthcare services are not addressed in Africa. Migration, climate change and fair trade (with their consequence on health) must also be addressed in an integrated way to ensure that Africans do not remain victims of global inequity.
- We welcome the commitment by the World Health Organization (WHO) to work with WONCA, the World Organisation of Family Doctors, through the Memorandum of Understanding signed on 28 January 2019. We are very encouraged by the active relationship established between WHO and WONCA in Africa at this conference and will support WHO AFRO in its quest to leave no one behind in Africa. However, we are concerned that there is limited commitment by African governments to include family doctors in the PHC team and to train the PHC team in family medicine.

We urge the United Nations General Assembly high-level meeting (UN HLM) on UHC in September 2019 in New York to make practical commitments to invest in PHC as a priority, especially on service delivery reforms that include the family doctor in the PHC team in Africa. Our desire is person- and people-centred primary healthcare.

Issued by WONCA Africa (www.WONCAAfrica.org)

Kampala 08 June 2019.

Supported by:

- WONCA World
- Primafamed Network
- The Besrour Centre at the College of Family Physicians of Canada
- Association of Family Physicians of Uganda
- Association of General and Private Medical Practitioners of Nigeria
- College of Primary Care Physicians of Zimbabwe
- Faculty of Family Medicine, National Postgraduate Medical College of Nigeria
- Kenya Association of Family Physicians
- Lesotho Medical Association
- Society of Family Physicians of Nigeria
- South African Academy of Family Physicians
- The Society of Family Physicians of Ghana
- West African College of Physicians Ghana Chapter Faculty of Family Medicine
- AfriWon (Young Family Doctor Movement - Africa)
- WONCA Working Party for Women in Family Medicine (Africa)
- Faculty of Health Sciences, Lúrio University, Nampula, Mozambique
- SHE Collaborates, Maastricht University
- WHIG (Dutch Taskforce on General Practice and International Health)
- Faculty of Medicine and Health Sciences of Stellenbosch University
- Ukwanda Centre for Rural Health, Stellenbosch University
- The Department of Family and Community Medicine, University of Gezira
- Global Familymed Foundation from Canada
- Aga Khan University Medical College, East Africa
- Bungham University College of Medicine and Health Sciences, Jos, Nigeria
- Family Medicine Department, College of Medical Sciences, University of Jos, Plateau State, Jos, Nigeria
- Family Medicine Unit, Department of Community Medicine, University of Ibadan, Ibadan, Oyo State, Nigeria
- Department of Family Medicine, Faculty of Clinical Sciences, College of Health Sciences, University of Uyo, Uyo, Nigeria
- Department of Family Medicine, College of Health Sciences, University of Ilorin
- Department of Family Medicine and Rural Health, Walter Sisulu University
- Medcamer, Médecins du Cameroon
- Department of Community & Family Medicine, University of Zambia
- Primary Health Care Directorate, University of Cape Town
- Namibian Academy of Family Physicians
- Dept. of Family and Community Medicine, University of Namibia
- Department of Family Medicine and Public Health Medicine, Faculty of Medicine, University of Botswana
- Department of Family Medicine & Primary Health Care, Protestant University of Congo
- Lesotho Boston Health Alliance (LeBoHA)
- Boston University Institute for Health Systems Innovation and Policy
- Center for Global Health, Aarhus University, Denmark
- Department of Primary Health Care, University of Rwanda

